# SWEDISH TRANSLATION, CULTURAL ADAPTATION AND TESTING OF THE PROSTHETIC UPPER EXTREMITY FUNCTIONAL INDEX-2

**DOI:** 10.2340/jrm-cc.v8.42151

**Published:** 2025-06-09

**Authors:** Cathrine WIDEHAMMAR, Lis SJÖBERG

**Affiliations:** 1 School of Health Sciences, Örebro University, Örebro, Sweden; 2 Department of Prosthetics and Orthotics, Örebro University Faculty of Medicine and Health, Örebro, Sweden

**Keywords:** upper limb prosthetics, outcome measure, occupational therapy, paediatrics, rehabilitation

## Abstract

**Objective:**

We aimed to translate, culturally adapt and test the Prosthetic Upper Extremity Functional Index-2 for a Swedish context.

**Subjects:**

Ten children with congenital upper limb deficiency with an upper limb prosthesis and their parents.

**Methods:**

The translation and cultural adaptation of the Prosthetic Upper Extremity Functional Index-2 was conducted according to the International Society for Pharmacoeconomics and Outcomes Research Principles of Good Practice for cross-cultural adaptation of patient-reported outcome measures; this comprises 10 steps, including Preparation, Forward Translation, Reconciliation, Back Translation, Back Translation Review, Harmonization, Cognitive Debriefing, Review of Cognitive Debriefing Results and Finalization, Proofreading and Final Report.

**Result:**

The new translated version, tested on 10 children, 4 boys and 6 girls, 3–14 years showed good relevance for the Swedish context, the questions were easy to understand, and response options were easy to interpret. It was also easily accessible on computers and mobile devices.

**Conclusion:**

The Swedish version of the Prosthetic Upper Extremity Functional Index-2 is user-friendly and provide information of the child’s self-reported prosthesis use in a Swedish context. Children’s right to express their opinions, is crucial, and using the questionnaire prior to their clinic visits gives children the opportunity to participate in goal setting and treatment planning.

## LAY ABSTRACT

The Prosthetic Upper Extremity Functional Index (PUFI) is a Canadian self-report questionnaire for children (3–18 years) with an upper limb prosthesis. The questionnaire asks about performance of various 2-handed activities. Children under 10 years answer with a parent. The answers provide a comprehensive picture of the child’s real-world prosthesis use. Children’s daily activities change with time, and therefore an updated Canadian version, PUFI-2, has been developed. We aimed to translate and culturally adapt PUFI-2 for use in a Swedish context. The Swedish version was tested on Swedish children and parents who stated that the questions were relevant, and easy to understand and respond. The PUFI-2 gives children the opportunity to participate in goal setting and treatment planning.

Children with upper limb reduction deficiencies, congenital or acquired, use their residual limb, prosthesis or assistive devices to perform daily activities. With the ability to operate an upper limb prosthesis and integrate it in everyday activities, the prosthesis helps the child relieve the load on the intact arm and hand and enables the performance of bimanual activities. Research shows that fitting an upper limb prosthesis at an early age benefits future prosthetic use (1–3). The recommendation is to start with a passive prosthetic fitting at about 6 months of age (1, 4) with the transition to an active (conventional body-powered or myoelectric) prosthetic hand between 2½ and 4 years of age (3).

Despite the benefits of prostheses, children sometimes choose not to use them, for various reasons (5). The prosthesis may be perceived as a hindrance, which needs to be investigated by the prosthetic treatment team. One important tool in this investigation is the availability of a self-report instrument that identifies the child’s everyday use of the prosthesis and its benefits in performing age-appropriate daily activities. However, there is a lack of clinical self-report instruments that reflect children’s perspective on prosthesis use and its pros and cons in their performance of daily activities. Examples of older self-report instruments are the 3 versions of The Child Amputee Prosthetics Project, Functional Status Inventory, for children of different ages (6–8). All 3 instruments record the frequency of performing an activity and whether a prosthesis is used or not. The child’s own perspective is limited since all versions use parents as proxies.

The Children’s Hand-use Experience Questionnaire, CHEQ 2.0 (9), is a web-based questionnaire that measures how children with reduced hand function experience their hand function in various daily activities. CHEQ 2.0 is focused on fine motor skills, thus some everyday activities, such as cycling, are not included, and the questionnaire has not been validated for prosthesis users. A more comprehensive web-based questionnaire, valid for prosthesis users, is the PUFI (10). PUFI is a Canadian parent-report and child-report questionnaire, which asks about the performance of different 2-handed, age-related daily activities within the following activity categories: Self-care, Home, School, Leisure, Community. It provides a comprehensive picture of a child’s real-world prosthesis use, and children as young as 10 years can self-report (10). PUFI covers different aspects of health according to ICF and is therefore recommended as an outcome measure for children fitted with an upper limb prosthesis (11). However, daily activities for children change with time, and therefore an updated Canadian version has been developed, PUFI-2. The number of activities has been reduced and some of the activities have been replaced. The updated version needs to be validated, and in order to be part of this validation we aimed to translate and culturally adapt PUFI-2 for use in a Swedish context.

## METHODS

### Instrument versions

*The original Prosthetic Upper Extremity Functional Index.* The original PUFI was developed by a clinical research group at Holland Bloorview Hospital, in Toronto, Canada, to evaluate the extent to which a child actually uses a prosthesis for daily activities, for example, the ease of activity performance with and without the prosthesis. Three types of validity of PUFI have been investigated: discriminant validity (12), criterion validity (12) and construct validity (12, 13).

PUFI has demonstrated acceptable discriminant validity, distinguishing prosthetic skill and use patterns between children of different ages and across the functional activities (12). Criterion validity was evaluated through comparisons of parent-report PUFI responses with an assessor’s scores of a child’s observed performances of PUFI activities, and Spearman’s rank correlations showed moderate correlation, weighted ƙ range 0.44–0.65 (12). Concerning construct validity, Spearman’s rank correlations showed a moderate-to-strong correlation for the categories “ease of performance” and “usefulness of prosthesis”, with a weighted ƙ range of 0.22–0.82 (12), and ƙ 0.82 (13) and good correlation between prosthesis wearing time and PUFI scores, weighted ƙ 0.70 (13).

The test-retest reliability of PUFI has been evaluated by repeated testing at different time points (10, 13), showing acceptable-to-excellent intra-class correlations (0.40–0.89), and inter-rater reliability between child and parent showed intra-class correlations in the range 0.30–0.77 (10, 12).

*The Prosthetic Upper Extremity Functional Index version 2, PUFI-2.* PUFI-2 is a web-based questionnaire under development that can be completed either at home or at the clinic. Children from 10 years can self-report, while for younger children the parents report. PUFI-2 has 2 age versions and is designed to be used throughout the child’s developing years. The younger child version, for children aged 3 to 6 years, contains 23 activities (items) classified into 4 activity categories, *Self-care, Home, Leisure, Community*, and for older children, aged 7 to 18 years, there is a parent-report version and a self-report version, both containing 27 activities (items) classified into 5 activity categories, as above but with the additional category *School*. All the PUFI-2 items concentrate on the performance of 2-handed activities that require either the use of the prosthetic device in an active or passive capacity or the use of the residual limb if the child is not wearing a prosthesis. For each item, 5 questions, covering (*i*) actual performance of the activity, (*ii*) method of performance, (*iii*) ease of prosthetic use, (*iv*) perceived usefulness of the prosthesis and (*v*) ease of performance without the prosthesis, are answered on an ordinal scale. See Table I for the questions and response options.

**Table I:** Description of the original PUFI (2021) and the revised PUFI-2 (2023)

Questionnaire content	PUFI		PUFI-2	
Versions and activity categories	Young child 3–6 years, parent-report	26 activities/items Activity categories: *dressing* 7 *self-care* 0 *school/play* 11 *extracurricular* 5	Young child 3–6 years, parent-report	23 activities/items Activity categories: *self-care* 5 *home* 1 *leisure* 13 *community* 4
	Older child 7–18 years, self-report and parent-report	38 activities/items Activity categories: *dressing* 8 *self-care* 4 *domestic* 10 *school/play* 6 *extracurricular* 10	Older child 7–18 years, self-report and parent-report	27 activities/items Activity categories: *self-care* 6 *home* 11 *school* 3 *leisure* 6 *community* 1
Response option sets	**Four sets** of response options were developed for each item/activity, designed as ordinal scales from the highest to lowest degree of function or competence.		**Five sets** of response options were developed for each item/activity, designed as ordinal scales, as for PUFI.	
Question			***Do you do this activity? /*** ***Does your child do this activity?***	
Response options			# Yes # Has not tried but could probably do it ^a^ # No, cannot do it even with help ^a^	
Question	***How do you usually do the activity?***		***How do you usually do the activity? /*** ***How does your child usually do the activity?***	
Response options	# Both arms together with the prosthetic hand or hook used actively to grasp the [specific object indicated here] # Both arms together with the prosthesis used passively to position or stabilize the [specific object indicated here] # With assistance of the residual limb # Non-prosthetic hand alone # With some help from another person # Cannot do # Never need to do it or am too young to do it		# Both arms together with the prosthetic hand or terminal device used actively (open and close hand/device to hold the object) # Both arms together with the prosthesis used passively (to position or stabilize the object, hand does not open/close) # With assistance of residual limb and/or another body part and/or other assistive devices # With non-prosthetic hand alone # With some help from another person # Don’t know/not sure ^a^	
Question	***How well do you do the activity with the prosthesis?***		***How well do you do the activity using the prosthesis? /*** ***How well does your child do the activity using the prosthesis?***	
Response options	# With no difficulty # With some difficulty # With great difficulty # With some help from another person # Cannot do it with the prosthesis		# With no difficulty # With some difficulty # With great difficulty # It is so difficult that I /my child need/ needs help from another person # Even with help, I /my child cannot do it using the prosthesis	
Question	***How useful is the prosthesis for the activity?***		***How useful is the prosthesis for the activity?***	
Response options	# Very useful # Somewhat useful # Not useful		# Very useful # Somewhat useful # Not useful	
Question	***How well do you do the activity without the prosthesis?***		***How well do you do the activity without the prosthesis? /*** ***How well does your child do the activity without the prosthesis?***	
Response options	# With no difficulty # With some difficulty		# With no difficulty # With some difficulty # With great difficulty # It is so difficult that I/my child need/needs help from another person # Even with help, I/my child cannot do it without the prosthesis.	

PUFI: Prosthetic Upper Extremity Functional Index.

a With follow-up questions about imagining how the activity could be performed.

The response options, structure and scoring of the younger and older child versions of PUFI-2 are the same. If the child has not performed the activity, the respondent (child or parent) is prompted to try to imagine how the child would do it, the ease of performance and how useful they think the prosthesis would be for that activity. To facilitate for children and parents, PUFI-2 has an instructive introductory video, and all items and response options are illustrated with descriptive photographs. The result is visualized through pie charts showing the proportion of each response option, 1 pie chart for each of the 5 questions.

In addition to the pie chart, the responses to the question *Does the child do the activity?* are also summarized per activity category in a stacked bar chart. The stacked bars show sum scores for the 3 response options: (*i*) *Yes (can perform)*, (*ii*) *Has not tried* and (*iii*) *Cannot do it*.

A total score is also calculated for the last 3 questions about ability with and without the prosthesis and prosthesis usefulness. The different total scores are calculated as follows:

Ability With Prosthesis – no difficulty ×4, some difficulty ×3, great difficulty ×2, needs help ×1, cannot do it ×0. Divided by total possible score = number of items ×4 (no difficulty)

Prosthesis Usefulness – very useful = ×2, somewhat useful = ×1, not useful = ×0. Divided by total possible score = number of items ×2 (very useful)

Ability without prosthesis – no difficulty = ×4, some difficulty ×3, great difficulty 2, needs help ×1, cannot do it ×0. Divided by total possible score = number of items ×4 (no difficulty).

### The translation process and cultural adaptation of PUFI-2

The translation process and cultural adaptation was conducted according to the International Society for Pharmacoeconomics and Outcomes Research (ISPOR) guidelines *Translation and Cultural Adaptation of Patient Reported Outcomes Measures – Principles of Good Practice* (14). The 10-step procedure includes Preparation, Forward Translation, Reconciliation, Back Translation, Back Translation Review, Harmonization, Cognitive Debriefing, Review of Cognitive Debriefing Results & Finalization, Proofreading and Final Report.

*Preparation.* The first author had been invited by the original developers of PUFI in Canada to take part in updating PUFI into a new version with activities more adapted to children in the 2020s. To be able to test the new version, PUFI-2, in Sweden, a translation to Swedish was needed. Preparation started by obtaining translation tables for the 3 different versions of PUFI-2 and the information video script from the Canadian research team.

*Translation, reconciliation, review and harmonization.* The forward translation was made by 3 individual translators, all native speakers of the target language, Swedish, and occupational therapists with experience from rehabilitation of children with upper limb deficiencies. All 3 versions of PUFI-2 were translated: *younger child parent-report*, *older child parent-report* and *older child self-report*. All included the questions, response guide, a preamble, description of the activities, explanation of buttons and prompts (since the questionnaire was web-based). A script for an information video about PUFI-2 was also translated. During the reconciliation phase, the 3 individual translated versions were discussed until consensus was reached on a single forward translation for each of the 3 PUFI versions and for the information video script.

The back translation to the original language, English, was performed by a bilingual expert to ensure both conceptual and semantic equivalence. To perform a back translation review the translation tables were sent to the Canadian developers of PUFI and PUFI-2, and the cultural and linguistic adaptations were discussed with them to ensure the conceptual equivalence of the translation. See Table II for examples of the cultural and linguistic adaptations. Appendix S1 contains a complete list of all the adaptations made. Since no other translation of PUFI had been published, the *harmonization* with other language translations and cultural adaptations of PUFI-2 was made in collaboration with the Canadian developers, who had contact with for example Dutch translators.

**Table II:** Examples of translation decisions

Original English version	Swedish version	Back translation	Translation decisions
Terminal device	Typ av proteshand	Type of prosthetic hand	*There is no Swedish term for “terminal device.” Instead, the description “type of prosthetic hand” is used.*
Upper limb	Arm	Arm	*“Upper limb” is a medical term that children and parents do not understand. In Sweden the name of the limb (arm or leg) is used instead of upper or lower limb.*
Examples include a myoelectric hand, a hook or a specific device used for certain activities (e.g. riding a bike, gymnastics etc.)	Myoelektrisk hand, passiv hand, krok eller protes utformad för specifik aktivitet, (ex simning, skidåkning, gymnastik)	Myoelectric hand, passive hand, hook, or prosthesis designed for a specific activity (e.g. swimming, skiing, gymnastics)	*We have added a device that is often used in Sweden, the passive hand prosthesis.* We also added *common activities where Swedish children use devices: swimming and skiing. A bicycle prosthesis is not used in Sweden and has been removed.*
Peel back the cover of a snack pack (e.g. cheese, hummus, jam)	Dra av locket från en liten förpackning med smör, mjukost, marmelad.	Remove the lid from a small container of butter, soft cheese, or jelly.	*Butter is usually found in portion packs and is more common than hummus in Sweden.*

*Cognitive debriefing – test of Swedish PUFI-2.* To examine understandability, interpretation and cultural relevance of the translation, the Swedish PUFI-2 was tested on a small group representing the target population, that is children age 3–18 years with upper limb prosthesis. The original developers incorporated the Swedish version of PUFI-2 into the REDCap digital platform and made it available for testing. Children and parents who visited the clinic were selected consecutively to cover all 3 age-versions of PUFI-2. They received both oral and written information about the test of the Swedish version and all agreed to participate. All participants got a link to the REDCap platform and completed the PUFI-2 questionnaire from home, on a computer or smartphone. Shortly after the questionnaire had been completed, a cognitive debriefing in the form of a structured interview was conducted with the child and/or the parents, during their visit at the prosthetic clinic or via an online video meeting. All interviews were performed by the first author (CW), who used a structured interview protocol and took notes during the interviews. The interview started with demographic questions, for example level of amputation and self-reported prosthesis use. For details in the structured interview protocol, please see the Appendix S1 and Table III Characteristics of the participants testing the Swedish PUFI-2. The interview continued with questions about how easy or hard it was to read and understand the PUFI-2 questions and response options. The participants were asked whether the activities were things that the child normally did and, if not, what activities were missing. The protocol also included questions about the feasibility of the digital format and open-ended questions such as ‘What is your overall experience answering PUFI-2?’ and ‘Do you have any additional comments about the questionnaire?’ Finally, a summary of the individual PUFI-2 result was presented for each participant.

**Table III:** Characteristics of the participants testing the Swedish PUFI-2

Child boy/girl	Age years	Level of amputation	Prosthetic side	Prosthesis use*	PUFI version	Parent/self-report
Boy	3	trans-carpal	right	Occasional	Young	Parent-report
Boy	3	trans-carpal	left	Half-day	Young	Parent-report
Girl	4	trans-radial	left	Daily	Young	Parent-report
Girl	4	trans-radial	right	Occasional	Young	Parent-report
Girl	8	trans-radial	left	Daily	Older	Parent-report
Girl	9	trans-radial	right	Daily	Older	Parent-report
Boy	11	trans-carpal	left	Half-day	Older	Self-report
Girl	13	trans-humeral	right	Half-day	Older	Self-report
Boy	13	trans-carpal	left	Half-day	Older	Self-report
Girl	14	trans-radial	right	Half-day	Older	Self-report

PUFI: Prosthetic Upper Extremity Functional Index.

* Self-reported level of prosthesis use.

Daily = Uses prosthesis 8 h a day or more 7 days/week.

Half-day = Uses prosthesis 4 h a day 7 d/week or 4–8 h a day 5 d/week (not weekend)

Occasional = Uses occasionally for a specific activity for example biking.

*Review of cognitive debriefing, proofreading and final report.* The answers from the cognitive debriefing were reviewed in order to notice discrepancies between the target groups interpretations of the translated questions and the original version of PUFI-2. After proofreading the Swedish PUFI-2, a final report (this article) was written to document the development of each translation. This includes a full description of the methodology used, and an appendix with an item-by-item representation of all translation decisions undertaken throughout the translation process, which can be used in future harmonizations of other language versions of PUFI-2.

## RESULTS

### Result of the translation process

After discussions with the original developers of PUFI-2, consensus was reached on the final version of the Swedish PUFI-2. During the translation, the most discussed problem area was the difficulty of finding corresponding Swedish words for the medical terms *terminal device*, *upper limb* and *residual limb*. Furthermore, in Sweden we do not use the word *clinician* when talking to children. Instead, we clarified that it was a person from the dysmelia treatment team. Another issue was the different types of prostheses and assistive devices. The following commonly used Canadian prostheses are rarely used in Sweden: *the body-powered hook, cosmetic prosthesis* and *bicycle prosthesis*. Those words were replaced with more relevant Swedish alternatives, such as *passive prosthetic hand* and *prosthesis for skiing*.

Most activities are the same for children in Sweden and Canada, but some cultural adaptations were needed, for example, children in Sweden play “brännboll” (*rounders/baseball*) instead of *cricket*. Other cultural differences were children’s *clothes* and *eating habits*. For example, small Swedish children often wear *tights*, which was not a Canadian alternative, but the original developers agreed that we could add *tights* in the Swedish version. We also had to adapt the bread spreads for the children’s snacks to more Swedish alternatives. Some words were related to the digital format used and had to be explained in the Swedish version. For examples of item-by-item description of translation decisions, see Table II. For a full item-by-item representation of all translation decisions undertaken throughout the process, see Appendix S1.

### Result Cognitive debriefing – test of Swedish version PUFI-2

Four boys and 6 girls aged from 3 to 14 years tested the Swedish version. The children had different levels of amputation, a myoelectric arm prosthesis from Ottobock, and they reported different levels of prosthesis use, ranging from occasional use to daily use. All 3 PUFI-2 versions were tested (younger child parent-report, older child parent-report and older child self-report). For the children under 10 years, the parents completed the relevant parent-report version. See Table III for the demographic characteristics of the participants in the testing. Overall, the distribution of test responses showed that no items were missing and that all response options were used.

All 10 children and their parents participated in the structured interviews about the understandability, interpretation and cultural relevance of the translation. Overall, the questions were easy to understand, the answer option easy to interpret and the items had cultural relevance. A summary of the answers is presented in Table IV.

**Table IV:** Structured interview protocol showing the result of the cognitive debriefing

Questions	Responses: A summary of responses from all participants			
	Very easy	Easy	Difficult	Very difficult
How was it to read and understand the questions?	6	4	0	0
If you answered difficult or very difficult, explain what was difficult?	No comments			
How was it to read and understand the response options?	3	7	0	0
If you answered difficult or very difficult, explain what was difficult?	No comments			

	All of them	Many of them	A few	None

Were the 23/27 activities things that you/your child usually does?	7	3	0	0
Did you miss any activity?			4	6
Which ones are not performed?	Tie shoes (Yong child version) Groome fingernail (Yong child version) (*Does with help*) Use scissors (Yong child version) Buttoning pants (Yong child version)			
What activities are you missing?	putting up one’s hair threading necklaces playing the trombone skiing playing floorball (Yong child version) do push-ups			
What is your overall experience answering PUFI-2?	It was fun! It was easy Easy to understand when you look at the pictures It was quick to respond It was a bit long Many questions, but it went fast Fun. It was smooth. Many questions but it went well Many questions, it took longer than I thought It did not take so long, and was not difficult			
How did you answer the questionnaire?	Computer 3 Phone 7			
How long did it take?	15–35 min (mean 25 min)			
Do you have any additional comments about the questionnaire?	The pictures give good help to understand It was hard to imagine how some activities might be performed Difficult to answer for another person Too many follow-up questions I think it’s easier and faster for the children to answer themselves Good that you could write comments to your answers Valuable to see the photos, how other children solved things The photos and the guide were good Stupid that you couldn’t interrupt and continue later Good with the pictures, got tips on solutions Makes it clear what you need to practice Good with a mix of activities, not just playing. Good to also map out useful activities, so they can practice them. I do things in different ways, and want to be able to choose multiple answer options for some of the questions My child is 3 and gross motor so he doesn’t do everything yet The response guide was great, clearly explained the different answer options			

PUFI: Prosthetic Upper Extremity Functional Index.

Furthermore, when the summary of the individual PUFI-2 result was presented to children and parents during the cognitive interview, all of them reported that the profile agreed with the child’s activity performance and real-world prosthesis use.

### Review of cognitive debriefing

The content of PUFI-2 was stated to be relevant for most of the participants, both in terms of the relevance of the activities and its cultural relevance. The presented activities were said to largely suit the children’s age and to be common for children in a Swedish context. Some activities, for example, grooming fingernails were perceived by parents to be too difficult for the smallest children. Despite this, the activities remain since children’s fine motor skills are developing differently. Activities that some respondents mentioned as missing, such as putting up one’s hair or playing the trombone, are activities that not all children necessarily do, and have therefore not been included. Some children and parents wanted the possibility to choose more than 1 response option depending on different circumstances. The single-choice option remains as *the most common way* of doing the activity is supposed to be stated and there is an option to comment the answer. The follow-up questions that required imagining possible scenarios of how an activity that the child does not usually do could be performed were perceived as not relevant and difficult to answer. According to the original PUFI developers these questions must remain, and it can be used in discussion with the therapist when planning future treatment. Another issue, the wish to pause and continue answering later, was however, something that the original developers were positive about changing.

## DISCUSSION

This study describes the methodological process for translation and cultural validation of the Canadian PUFI-2 from English into Swedish, performed according to the internationally established ISPOR guidelines (14). According to Wild et al. (14), a published report is essential for future translations of the same measure to be harmonized with language versions previously developed. Therefore, our detailed description of the process in this publication will be helpful when PUFI-2 is translated to create additional language versions.

The overall impression from participants who tested the Swedish version was positive. PUFI-2 was experienced as user-friendly, accessible on both computers and mobile devices, with relevant activities and with descriptive photographs illustrating the activities and alternative ways of performing them. It is recommended that self-report questionnaires for children are illustrated with photographs and well-tailored to the different age groups and contexts (15), which the participants in the test of the Swedish version also confirmed during the cognitive debriefing.

Some children and parents highlighted how difficult it was to choose just one way of performing an activity. This presumes that the child usually performs the activity in just one way; actively with the prosthesis, passively with the prosthesis, with the residual limb, with one hand only, or with help from someone else. Several of the participants pointed out that it depends on the situation. How they perform an activity is often determined by the social and physical context in which the activity is performed. The importance of the social context (16, 17) and the fact that prostheses use in general has not yet been fully established could be an explanation for why some of the children in our testing of Swedish PUFI-2 had difficulty deciding how they usually perform the activity. In addition, previous research has shown that the social context turns out to have a greater influence on the child’s prosthesis use than the functional demands of a particular grip (16, 18, 19).

The youngest children reported that some activities were not performed. They were gross motor and therefore used Velcro instead of shoelaces, didn’t use scissors or jeans with buttons. Due to large variations in development among the youngest children, we would like to advise some caution in introducing PUFI-2 too early, as the parents of a child with fine motor difficulties who answers the questionnaire may perceive low scores as a failure. Therefore, when using PUFI-2 in the clinic it is important to consider the child’s individual maturity level in relation to the questions asked.

A well-known issue is reporting by proxy. The challenge the parents who answered PUFI-2 had is well known through an extensive number of studies. For example, Sheffler et al. show that children with congenital upper limb deficiency report better upper-extremity function compared to their parents’ perceptions (20). This kind of disagreement is also found in a review by Hemmingsson et al. (21) with the summary that parents in general consider their child to have more extensive difficulties than the children themselves think they have. Despite these differences, self-report assessments that include both parents’ and children’s views are strongly recommended (21). We recommend parents to answer PUFI 2 together with their child, preferably before they visit the clinic, to provide the best possible basis for treatment planning.

It is important to consider children’s right to express their opinion, including in healthcare settings. According to the UN Convention on the Rights of the Child (22), every child has the right to express their opinion. By using PUFI-2 before the children visit the rehabilitation clinic, they have increased opportunities to take part in goal setting and planning of their own treatment. Earlier research highlights the importance of capturing the children’s own perspective on prosthesis use, on their own ability and the performance of daily activities, as well as the need for validated and stable instruments to provide this information (5, 17, 23). We believe that PUFI-2 is a good choice for taking the child’s perspective into account, but this needs to be confirmed by psychometric testing of validity and reliability.

### Methodological considerations

The recommended sample size for testing a translated version on the target group is 5–8 (14). We decided to include 10 children to cover all ages, 3–18, for the different PUFI-2 versions. The oldest child included was 14 years old, which gave limited evidence of how the questionnaire is perceived by children in their upper teens, 15–18 years old. The trustworthiness of the study is, however, ensured by the varied sample with both boys and girls of different ages, with different experiences of prosthesis use and different habits of use. Furthermore, by following the structured ISPOR procedure for translation and cultural validation (14) and the thorough documentation of the method used, also assures trustworthiness. Moreover, the 3 different versions of forward translations made by experts from the field, and the use of a bilingual expert for backward translation, ensure the conceptual and semantic equivalence in wordings according to the original instrument, which contributes to increase the trustworthiness of this study. A possible limitation is the harmonization process, which only was verbally agreed upon with the original developers, since there are a few other language translations to compare with, but none is published yet. This highlights the importance of this publication, to facilitate future languages translations.

In conclusion, the Swedish PUFI-2 version is easy to understand and interpret and has cultural relevance for a Swedish context. By adding the function *pause and continue later*, PUFI-2 will be even more user-friendly, for today’s busy parents and children with less amount of patience. Impatience or not, children’s right to express their opinions, even in healthcare settings, is important. Using PUFI-2 prior to their clinic visits allows children to participate in goal setting and treatment planning. PUFI-2 is also a helpful tool for the multidisciplinary team (therapists and prosthetist), by enabling both evaluation of prosthesis prescription and of the performance of daily activities, regardless of how much the prosthesis is used. Future PUFI-2 research will include a more extensive sample with children of all ages and psychometric testing of validity and reliability.

## Supplementary Material

SWEDISH TRANSLATION, CULTURAL ADAPTATION AND TESTING OF THE PROSTHETIC UPPER EXTREMITY FUNCTIONAL INDEX-2
