# Supplementary material for: SWEDISH TRANSLATION, CULTURAL ADAPTATION AND TESTING OF THE PROSTHETIC UPPER EXTREMITY FUNCTIONAL INDEX-2
Source: J Rehabil Med Clin Commun. 2025 Jun 9;8:42151. doi: 10.2340/jrm-cc.v8.42151 (PMC12171865; doi:10.2340/jrm-cc.v8.42151)
Supplement: SWEDISH TRANSLATION, CULTURAL ADAPTATION AND TESTING OF THE PROSTHETIC UPPER EXTREMITY FUNCTIONAL INDEX-2 [file JRMCC-8-42151-s1.pdf]

Supplementary material has been published as submitted. It has not been copyedited, or typeset by Journal of Rehabilotation Medicine – Clinical Communications

Supplementary file: Structured interview protocol

Date: ID:

**Questions:**	**Response options:**				
**Demografic questions**					
Completed PUFI-version	Young child		Older child		
Parent/ self-report	Parent-report		Self-report		
Child (sex)	Boy		Girl		
Age (years)					
Level of amputation	Trans humeral	Trans radial	Trans carpal		
Prosthetic side	Left		Right		
Prosthesis	Myoelectric	Cosmetic	Activity specific	Other type	
Prosthetic use*	Daily	Half-day	Occasional	Sporadic	Never
**Questions about responding PUFI-2**					
How was it to read and understand the questions?	Very easy	Easy	Difficult	Very difficult	
If you answered difficult or very difficult, explain what was difficult?	Comments:				
How was it to read and understand the response options?	Very easy	Easy	Difficult	Very difficult	
If you answered difficult or very difficult, explain what was difficult?	Comments:				
Were the 23/27 activities things that you/your child usually does?	All of them	Many of them	A few	None	
Did you miss any activity?	All of them	Many of them	A few	None	
Which ones are not performed?	Comments:				
What activities are you missing?	Comments:				
What is your overall experience answering PUFI-2?	Comments:				
How did you answer the questionnaire? Computer/phone/tablet	Comments:				
How long did it take?	Comments:				
Do you have any additional comments about the questionnaire?	Comments:				

*Self- reported prosthetic use, explanation of response options:

Daily use = Uses prosthesis 8 hours a day or more 7 days/week

Half-day use = Uses prosthesis 4 hours a day 7 days/week or 4-8 hours a day 5 days/week (not weekend)

Occasional use = Uses occasionally for a specific activity, e.g. biking

Sporadic use = Uses sporadically some time every month, randomly

Never use = Has not used the prosthesis the last weeks, do not use prosthesis, or doesn't have a prosthesis that fits well
